# Ongoing transmission of lymphatic filariasis in Samoa 4.5 years after one round of triple-drug mass drug administration

**DOI:** 10.1371/journal.pntd.0012236

**Published:** 2024-06-27

**Authors:** Helen J. Mayfield, Benn Sartorius, Sarah Sheridan, Maddison Howlett, Beatris Mario Martin, Robert Thomsen, Rossana Tofaeono-Pifeleti, Satupaitea Viali, Patricia M. Graves, Colleen L. Lau

**Affiliations:** 1 University of Queensland Centre for Clinical Research, The University of Queensland, Brisbane, Queensland, Australia; 2 School of Public Health, Faculty of Medicine, The University of Queensland, Brisbane, Queensland, Australia; 3 Samoa Ministry of Health, Apia, Samoa; 4 School of Medicine, National University of Samoa, Apia, Samoa; 5 Oceania University of Medicine Samoa, Apia, Samoa; 6 College of Public Health, Medical and Veterinary Sciences, James Cook University, Queensland, Australia; University of Agricultural Sciences and Veterinary Medicine Cluj-Napoca, Life Science Institute, ROMANIA

## Abstract

**Background:**

Lymphatic filariasis (LF) remains a significant global issue. To eliminate LF as a public health problem, the World Health Organization (WHO) recommends multiple rounds of mass drug administration (MDA). In certain scenarios, including when elimination targets have not been met with two-drug MDA, triple-drug MDA (using ivermectin, diethylcarbamazine and albendazole) is recommended. In this study, we report on antigen (Ag) and microfilaria (Mf) prevalence in eight primary sampling units (PSUs) in Samoa 4.5 years after one round of triple-drug MDA.

**Methodology:**

In 2023, community surveys were conducted in eight PSUs that had been surveyed previously in 2018 (between 1.5 and 3.5 months post triple-drug MDA) and 2019 (six to eight-months post triple-drug MDA). Fifteen houses were randomly selected in each PSU with household members aged ≥ 5 years invited to participate. Blood samples were tested for Ag and Mf.

**Principal findings:**

Ag-positive participants were observed in six of the eight PSUs, and Ag prevalence was significantly above the 1% threshold in four PSUs. The presence of Mf-positive participants in five PSUs confirms the presence of residual active infections.

**Conclusions/Significance:**

This study provides evidence of persistent LF transmission in Samoa 4.5 years after one round of triple-drug MDA, confirming that one round was insufficient for interruption of transmission in this setting. Our findings highlight the negative impact of delaying MDA rounds, for example, due to public health emergencies.

## Author summary

The World Health Organization (WHO) recommends triple-drug mass drug administration (MDA) for the elimination of lymphatic filariasis (LF) as a public health problem for countries where targets have not been achieved using two-drug MDA, and where onchocerciasis is non-endemic. In 2018, Samoa was the first country to implement a national triple-drug MDA. This study surveyed 623 randomly selected participants across eight primary sampling units (PSUs) in Samoa, 4.5 years after the first round of triple-drug MDA. These results support the current WHO recommendations that multiple rounds of triple-drug MDA are required to interrupt transmission. A gap of 4.5 years between rounds of triple-drug MDA can result in a resurgence, with persistence of LF transmission and burden levels comparable to those observed before the MDA.

## Introduction

The Global Program to Eliminate lymphatic filariasis (GPELF) is one of the world’s largest public health programs and aims to eliminate this neglected tropical disease as a public health problem [1]. More than 9.3 billion treatments have been provided since the program began in 2000, treating more than 935.5 million people. Despite the program’s substantial progress in reducing the public health burden of lymphatic filariasis (LF) [1], 44 countries are considered as still needing further (or initial) mass drug administration (MDA) [2].

To eliminate LF from a population, multiple rounds of MDA are recommended to break the transmission cycle of the helminth parasite between humans and mosquitoes [3,4]. Delays between MDA rounds, not completing sufficient rounds, or poor coverage can reduce the effectiveness of MDA [5,6]. Failure to interrupt transmission can result in ongoing circulation or resurgence, meaning that countries risk losing any earlier gains made from many years of expensive, large-scale interventions [7,8]. Treatment during an MDA most commonly consists of diethylcarbamazine (DEC) and albendazole, or ivermectin and albendazole. In certain countries where two-drug MDA with DEC and albendazole has proven ineffective for LF elimination, triple-drug MDA (with the addition of ivermectin) is recommended [9–11]. Triple-drug MDA has been found to be well tolerated in terms of both social acceptance and safety [12–14], improve the effectiveness of MDA in some settings [4,15] and may decrease the number of rounds required to reduce microfilaria (Mf) levels below the target threshold for elimination [16].

In Samoa, LF remains endemic despite many rounds of MDA over recent decades [8]. The disease is caused by infection with the *Wuchereria bancrofti* parasite, transmitted primarily by the day-biting vector *Aedes polynesiensis* [17]. Disease transmission is diurnally sub-periodic meaning that Mf are present in peripheral blood at all times, although may be present in greater densities at certain times of day. While there is limited evidence suggesting that triple-drug MDA may not be more effective than two-drug MDA in areas of *Aedes*-transmitted LF [18], more work is required to better understand the potential benefits and limitations of the intervention.

In 2018, Samoa was the first country to roll-out a national triple-drug MDA [19]. The roll-out was largely successful in reaching the majority of the population, with a self-reported coverage of 80.2.% of the total population [19]. A second MDA round was scheduled for 2019, but was postponed due to a severe measles outbreak [20]. Further delays due to the COVID-19 pandemic meant the second round of triple-drug MDA was delayed until September 2023, five years and one month after the first round. The implications of this delay for national elimination efforts and LF persistence and/or resurgence are uncertain. This study reports on antigen (Ag) and Mf prevalence in eight primary sampling units (PSUs) in Samoa 4.5 years after one round of triple-drug MDA. In doing so, we aimed to establish the likelihood of ongoing LF transmission in the country.

## Methods

### Ethics statement

Ethics approvals were granted by the Samoan Ministry of Health and The University of Queensland Human Research Ethics Committee (protocol 2021/HE000895). The study was conducted in close collaboration with the Samoa Ministry of Health, the WHO country office in Samoa, and the Samoa Red Cross. Permission was sought from village leaders before entering a village. Verbal and written informed consent were obtained from all participants, or from the parents or guardians of participants under the age of 18 years.

### Study area

Samoa is located in the Pacific region and has a population of approximately 200,000 people [21]. The majority of residents live on the two main islands of Upolu and Savai’i, which consist of both urban and rural areas. This study reports on results from eight PSUs located on the main island of Upolu, covering both rural and urban settings.

### Survey design

In 2023 (4.5 years post-MDA), surveys were conducted from March 2–15 in eight PSUs (consisting of one or two villages each) selected from among 35 PSUs surveyed in previous studies in 2018 (1.5 to 3.5 months after the first round of triple-drug MDA) [22] and 2019 (six to eight months post triple-drug MDA) [23]. These previous studies were powered to detect a national prevalence of <2%, with a target sample size per PSU of 57 participants aged ≥10 years and 57 aged 5–9 years. These earlier surveys were designed to compare prevalence between the two age groups, and participants were recruited through household surveys of people aged ≥5 years, and convenience surveys of 5-9-year-olds [22]. In contrast, the 2023 survey was designed to report on observed Ag and Mf prevalence 4.5 years after one round of triple-drug MDA. The 2023 survey aimed to enrol a similar number of participants per PSU (~57) as in the household surveys from 2018 and 2019. A convenience survey of 5-9-year-olds was not conducted in 2023 because the study was not designed to compare prevalence between the two age groups.

**Fig 1:**
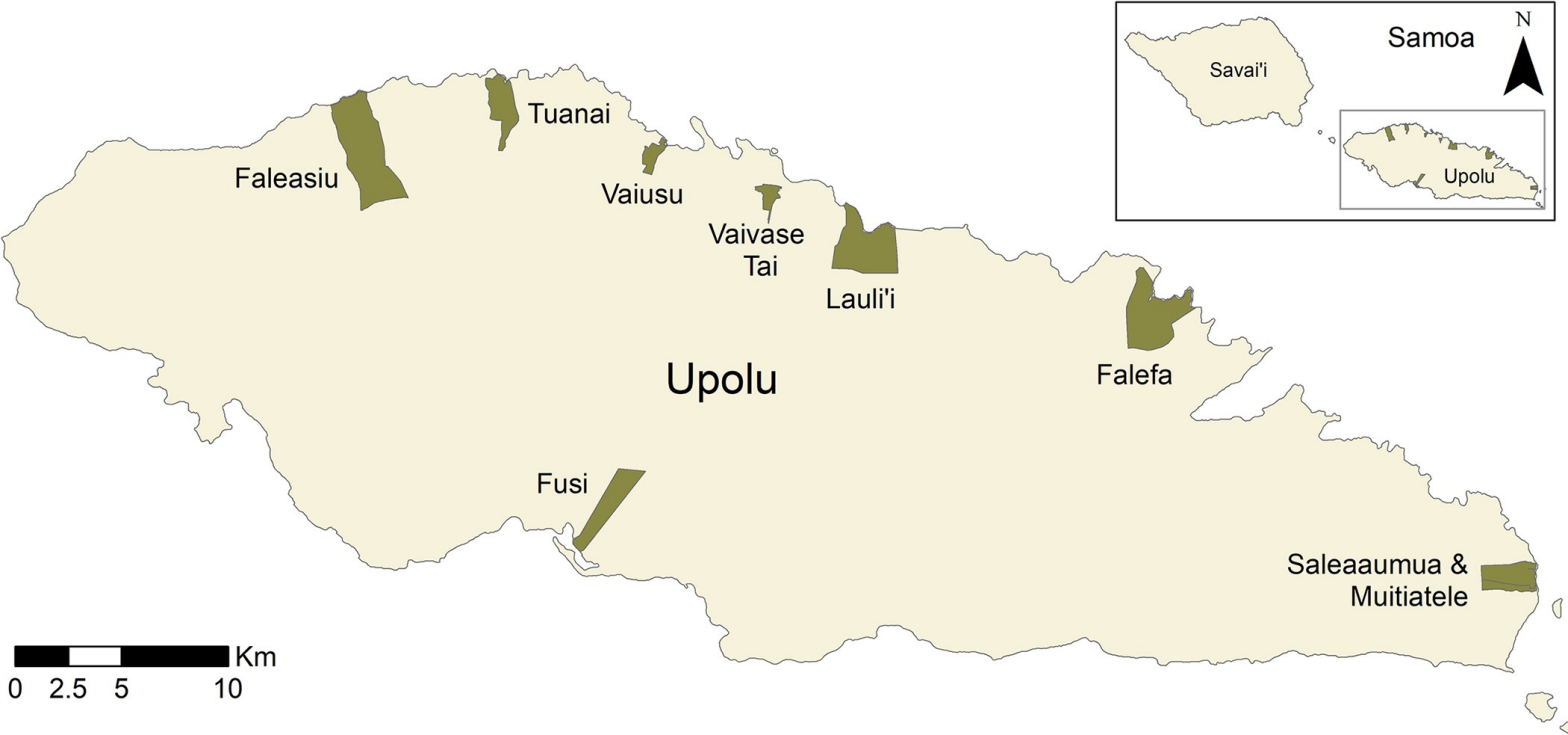
Location of primary sampling units (PSUs) selected for the 2023 Samoa survey on the island of Upolu, Samoa. Spatial data on country, island, region, and village boundaries in Samoa were obtained from the Pacific Data Hub (pacificdata.org accessed on 8 July 2020) and DIVA-GIS (diva-gis.org, accessed on 12 August 2019) under an open access licence available at https://pacific-data.sprep.org/resource/public-data-license-agreement-0.

PSUs for the 2023 survey were selected based on the estimated Ag prevalence observed in 2019. Selected PSUs are given in Table 1, along with the self-reported coverage of the population for the 2018 MDA [19]. To ensure a representative sample from the range of Ag prevalence settings, stratified sampling based on observed Ag prevalence in 2019 was used to select the PSUs (Table 1). Cut-offs for Ag prevalence categories were defined so that they were evenly distributed across the range of observed Ag prevalence values in 2019. Accordingly, we selected two PSUs with 0% Ag prevalence, and two each with low (3–4%), medium (6–7%), and high (13–16%) Ag prevalence observed in 2019 (Fig 1).

**Table 1:** Primary sampling units (PSUs) selected for the 2023 Samoa survey, including Ag prevalence in 2019 [23] and self-reported population coverage for the 2018 MDA (calculated as reported elsewhere [19]).

2019 Ag Prevalence	PSU	2018 MDA coverage % (95% CI)
None (0%)	Vaivase Tai	82.0% (73.1–88.1%)
	Mutiatele + Saleaaumua	87.2% 80.5–91.8%
Low (3–4%)	Tuanai	83.7% (77.7–88.4%)
	Fusi	87.3% (80.7–91.8%)
Medium (6–7%)	Vaiusu	72.1% (62.6–79.9%)
	Falefa	89.1% (80.1–94.3%)
High (13–16%)	Faleasiu	82.3% (78.6–85.4%)
	Lauli’i	65.6% (58.8–71.8%)

In each PSU, 15 houses were selected based on a virtual-walk method described previously [22,23]. Each selected household was visited at least once. If the selected building was not a residential dwelling, it was replaced with the nearest household, or the household of the building’s owner. Households that declined to participate were replaced with their nearest neighbour. Surveys were carried out between 2pm and 8pm from Monday to Saturday to maximise the number of people at home during the visit. If no one was at home at the time of scheduled field team visits, teams returned the following day where possible, or else replaced the household with a neighbour. Additional households were surveyed (selected at random) if the target sample size for that PSU had not been reached after visiting 15 households.

### Data and sample collection

For each participant, field teams used a smartphone with customised StandardData questionnaire (https://www.datastandard.co) to collect the household GPS location and conduct a demographic survey. Finger-prick blood samples (300–500 μL) were collected from each participant and stored in a cooler bag with ice packs until teams returned to the field laboratory. Samples were refrigerated overnight before testing, or until the following Monday if collected on a Saturday.

### Antigen and Mf testing

Blood samples were allowed to return to room temperature before being tested for Ag using Abbott Alere Filariasis Test Strips (FTS) (Scarborough, ME, USA). FTS were read at 10 minutes according to manufacturer’s instructions. For Ag-positive samples, up to three thick blood smears (slides) were prepared according to WHO guidelines [24]. Each slide had three 20μL lines of blood (i.e., 60 μL per slide). Slides were dried for 72 hours before being dehaemoglobinised in water for 5–10 minutes and dried again. Two of the three slides from each sample were then fixed with 100% methanol and stained with Giemsa according to WHO-recommended methods [24]. The third slide was left unstained as a backup or for future use. Slides were read initially in the field to identify Mf-positive individuals who needed treatment. On return to Australia, the two stained slides from each participant were read by independent readers (one reader per slide) to record Mf densities. Mf/mL for each Mf-positive participant was estimated by calculating the average number of Mf per 60 uL counted from each slide and converting to mL. Mf-positive participants identified in 2023 were offered treatment with ivermectin, DEC and albendazole, using the same weight-based dosage as the 2018 MDA.

### Statistical analysis

Although community surveys in 2018 and 2019 included both household survey and convenience survey components, only participants sampled from household surveys were included in the current analysis. This was done so that any comparison of results across the three years used data collected using a consistent sampling strategy and resulted in the inclusion of 558 participants across 105 households from 2018, and 643 participants across 122 households from 2019.

Survey weighted estimates of prevalence were conducted using the *Survey* package in R version 4.3.0 [25]. Prevalence estimates were weighted for sampling probability, i.e., the probability of selection of a household within a given PSU was calculated based on the total number of households sampled in the PSU divided by total number of estimated households within each PSU. Estimated houses per PSU were calculated from the 2016 National Census [26]. Prevalence estimates were standardised for age and gender distribution of the Samoan population based on the house and population distributions, also taken from the 2016 National census data [26].

To compare the Mf density for each Ag-positive sample, we calculated the geometric mean (due to skewed distributions) of the per mL Mf count for all Mf-positive participants in that year. The proportion of Ag-positive participants who were also Mf-positive in each year was calculated at the national level. Confidence intervals were calculated using the Clopper-Pearson method in the *BinomCI* function in the *DescTools* R package [27]. The change in Ag and Mf prevalence (with 95% confidence intervals) from 2018 to 2023 and from 2019 to 2023 was estimated using the formula provided in S1 Fig.

### Role of the funding source

The study funder had no role in study design, data collection, data analysis, data interpretation, writing of the report, or decision to submit the results for publication. The corresponding author had full access to all the data in this study and had final responsibility for the decision to submit for publication.

## Results

### Participants

In 2023, 623 participants were recruited from 125 households across the eight selected PSUs with a range of 58 to 101 participants per PSU. Participants’ ages ranged from five to 93 years (median 22 years). Demographics and sample size were similar in 2018, 2019 and 2023 (Table 2).

**Table 2:** Participant numbers and demographic details for 2018, 2019 and 2023 surveys in eight primary sampling units (PSUs) in Samoa, sorted by 2019 Ag prevalence category. Details of age and gender by PSU are provided in S1 Table.

			2018	2019	2023
Total participants			558	643	623
Sex n (%)		Male	275 (49.3%)	291 (45.3%)	280 (45.0%)
		Female	283 (50.7%)	352 (54.7%)	343 (55.0%)
Age (years)		Median (range)	24 (5–89)	20 (5–80)	22 (5–93)
Households (n)			104	121	125
Participants by PSU	2019 Ag	PSU			
	None (0%)	Vaivase Tai	60	58	61
		Mutiatele + Saleaaumua	71	89	68
	Low (3–6%)	Tuanai	69	76	72
		Fusi	68	69	91
	Medium (6–7%)	Vaiusu	68	100	101
		Falefa	80	74	58
	High (13–16%)	Faleasiu	70	102	72
		Lauli’i	72	75	100

### Antigen prevalence

**Fig 2:**
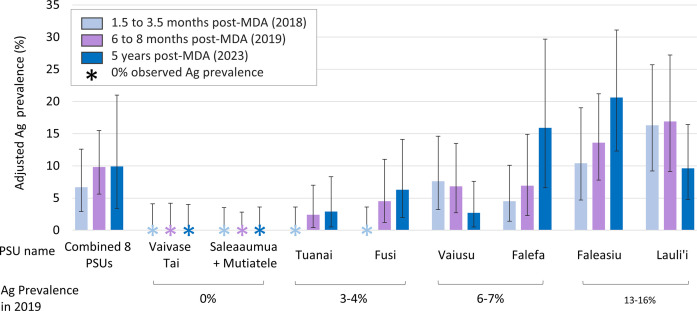
Antigen (Ag) prevalence for lymphatic filariasis in the eight selected primary sampling units (PSUs) in Samoa 1.5 to 3.5 months (2018), 6 to 8 months (2019) and 4.5 years (2023) after one round of triple-drug MDA in August 2018. Error bars show 95% confidence intervals (CI).

Overall adjusted Ag prevalence in the eight PSUs in 2023 was 9.9% (95% CI 3.5–21.0) compared with 6.7% (95% CI 2.9–12.6) in 2018 and 9.8% (95% CI 5.6–15.5) in 2019 (Fig 2 and S2 Table). There was no statistically significant change in the overall Ag prevalence of the eight selected PSUs from 2018 to 2023, or from 2019 to 2023. Ag-positive residents were identified in six of the eight PSUs with adjusted Ag prevalence in these ranging from 2.7% (95% CI 0.5–7.6%) to 20.6% (95% CI 12.2–31.8%). In 2023, no Ag-positive participants were identified in the two PSUs with zero Ag prevalence in 2018 and 2019.

Four of the eight PSUs showed an increasing trend in Ag prevalence from 2018 to 2023, although these changes were not statistically significant with the sample sizes for individual PSUs (Fig 2). Of the six PSUs where Ag-positive individuals were detected in 2023, change in prevalence from 2018 to 2023 ranged from an increase of 11.4% (95% CI 0.9–21.9%) in Falefa from 4.5% (95% CI 1.4–10.1%) to 15.9% (95% CI 6.6–29.7%), to a decrease of 6.7% (95% CI -17.0%- -3.6%) in Lauli’i from 16.3% (95% CI 9.2–25.7%) to 9.6% (95% CI 4.8–16.4%). Full details are given in S2 Table.

### Microfilaria prevalence

**Fig 3:**
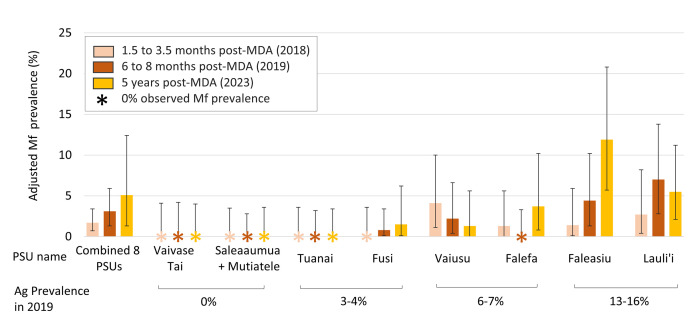
Microfilaria (Mf) prevalence for lymphatic filariasis in the eight selected primary sampling units (PSUs) in Samoa 1.5 to 3.5 months (2018), 6 to 8 months (2019) and 4.5 years (2023) after one round of triple-drug MDA in August 2018. Error bars show 95% confidence intervals (CI).

Overall Mf prevalence for the eight PSUs surveyed in 2023 was 5.1% (95% CI 1.3–12.4%). Full details of Mf prevalence at the PSU level are given in Fig 3 and S3 Table. Mf-positive individuals were identified in five of the six PSUs where Ag-positive participants were found in 2019. Ages for the 16 Mf-positive participants in 2023 ranged from 20 to 67 years (median 53 years). Fourteen (88%) Mf-positive participants were male and two (12%) were female. In 2023, geometric mean Mf density for Mf-positive participants was 108.9/mL (range 8.3 to 800.0), compared to 187.2/mL (range 16.7 to 1141.7) in 2018 and 121.0/mL (range 8.3 to 862.5) in 2019.

### Proportion of Ag-positive people testing Mf-positive

**Fig 4:**
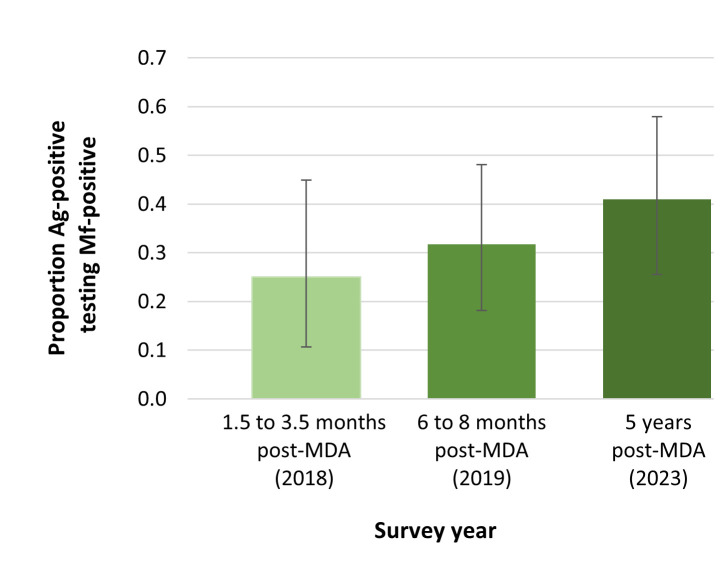
Proportion of Ag-positive participants who tested Mf-positive in six sentinel primary sampling units (PSUs) in Samoa 1.5 to 3.5 months (2018), 6 to 8 months (2019) and 4.5 years (2023) after one round of triple-drug MDA in August 2018. Error bars show 95% confidence intervals (CI).

Of the 39 Ag-positive participants in 2023, 41.0% (95% CI 25.5–57.9%) were Mf-positive, compared to 31.7% (95% CI 18.1–48.1%) of the 41 Ag-positive participants identified in 2019, and 25.0% (95% CI 10.7–44.9%) of 28 Ag-positive participants in 2018 (Fig 4).

## Discussion

Our 2023 survey of LF in Samoa detected residual infection from *W*. *bancrofti*, 4.5 years after one round of triple-drug MDA, as indicated by observed Mf in five of the eight PSUs. Overall Ag prevalence remained significantly above the 1% recommended threshold for stopping MDA, supporting WHO’s recommendation that at least two rounds of triple-drug MDA are required to interrupt transmission [24]. These findings provide evidence that one round of triple-drug MDA was not sufficient for sustained elimination of LF transmission in Samoa and highlight the impact of unplanned disruptions to elimination programs.

Despite the limited sample size, our current study confirmed that Ag prevalence was significantly above 1% (with lower 95% CI >1%) overall and in four of the eight PSUs. However, in the two PSUs where no Ag-positive participants were identified, we cannot conclude with confidence that Ag prevalence was below 1%. Although the increase from 2019 to 2023 in the ratio of Mf to Ag samples suggests ongoing transmission, the increase was not statistically significant and the results for the ratios should be considered as indicative rather than conclusive.

Triple-drug MDA was first distributed in Samoa in 2018. The second round was initially planned for mid-2019, but was delayed due to a severe measles outbreak [20], which necessarily became the priority for public-health resources and spending. From 2020, the public health focus on the COVID-19 pandemic further delayed the MDA roll-out. While our previous studies using molecular xenomonitoring of mosquitoes [23] demonstrated that LF transmission reduced from 2018 to 2019 (after one round of MDA), our current study shows that this single round of triple-drug MDA was not sufficient for sustained reduction over 4.5 years. This example from Samoa illustrates one of the key challenges for the GPELF, which aims to eliminate the public health burden of a chronic and mostly asymptomatic disease in the context of competing resources from more acute and high-priority health issues.

It is highly unlikely that study participants would have received treatment outside of the MDAs. It is therefore reasonable to assume that self-reported MDA participation rates in 2018, 1.5–3.5 months post-MDA, were reflective of community-level treatment rates. A previous study of MDA coverage in Samoa [19] observed that self-reported coverage of the 2018 MDA in the village of Lauli’i was lower than the overall national coverage. This suggests that infection prevalence would be less likely to decrease than in other villages. Contrary to expectations however, a decreasing although non-significant trend in both Ag and Mf prevalence from 2018/2019 to 2023 was observed in the village. Variation in actual prevalence between surveys depends on many factors including random variation, population mobility, behavioural factors (including MDA participation), vector control [28], and initial baseline infection levels [5,12]. Participant recruitment and sample size will also affect the degree to which the observed prevalence reflects the actual prevalence. We therefore urge that results by individual PSU should be interpreted with caution.

The timing of the surveys in relation to the MDA limits the conclusions that can be made regarding trends in Ag and Mf prevalence. The baseline survey in 2018 occurred between 1.5 and 3.5 months after the first round of triple-drug MDA, meaning that while Ag prevalence was not expected to have been affected, Mf prevalence would likely be lower than pre-MDA levels. The second survey in 2019, conducted six to eight months post-MDA, may not fully reflect the impact of MDA because Ag may persist for months after treatment [29]. Given this timing, and the four-year gap between the 2019 and 2023 surveys, it cannot be determined whether the Ag and Mf prevalence initially decreased followed by resurgence, or if the initial round of MDA was ineffective at reducing Ag and Mf prevalence. Molecular xenomonitoring of mosquitoes [23] in these PSUs in 2018 and 2019 indicate that the former is more likely, and that one round of triple-drug MDA likely reduced infection levels, but was insufficient to interrupt transmission in this setting.

*Aedes* mosquitoes are highly efficient vectors for the *W*. *bancrofti* parasite [30], potentially impacting the number and type of interventions needed to break the transmission cycle. The persistence of transmission may be partly related to the highly efficient *Aedes* vectors [31]. The diurnally sub-periodic transmission also presents unique considerations, particularly in relation to the locations where transmission is most likely to occur (i.e. school, home or workplace). These results may therefore be less applicable to settings with other species of vectors (*Culex or Anopheles*) and/or parasites (*Brugia*) [12]

In conclusion, the delays in delivering the second round of triple-drug MDA in Samoa have likely negated any gains from the first round delivered in 2018. This work provides evidence that a single round of triple-drug MDA is not sufficient for sustained interruption of LF transmission.

## Supporting information

S1 Fig Standard formulae used to calculate the lower and upper bounds for the delta (difference of two proportions). (PDF)

S1 Table Participant numbers and demographic details for 2018, 2019 and 2023 surveys in eight sentinel primary sampling units in Samoa. (PDF)

S2 Table Antigen (Ag) prevalence for lymphatic filariasis in the eight sentinel primary sampling units (PSUs) in Samoa in 2018, 2019 and 2023. (PDF)

S3 Table Microfilaria (Mf) prevalence for lymphatic filariasis in the eight sentinel primary sampling units (PSUs) in Samoa in 2018, 2019 and 2023. (PDF)
