# Supplementary material for: Ongoing transmission of lymphatic filariasis in Samoa 4.5 years after one round of triple-drug mass drug administration
Source: PLoS Negl Trop Dis. 2024 Jun 27;18(6):e0012236. doi: 10.1371/journal.pntd.0012236 (PMC11210818; doi:10.1371/journal.pntd.0012236)
Supplement: S1 Fig — (PDF) [file pntd.0012236.s001.pdf]

Ongoing transmission of lymphatic filariasis in Samoa 4.5 years after one round of triple-drug mass drug administration

Helen J Mayfield, Benn Sartorius, Sarah Sheridan, Maddison Howlett, Beatris Mario Martin, Robert Thomsen, Rossana Tofaeono-Pifeleti, Satupaitea Viali, Patricia M. Graves, Colleen L Lau

Supplementary S1

$$Lower\ bound = (\hat{p}_1 - \hat{p}_2) - z_{\alpha/2}\sqrt{\frac{\hat{p}_1(1-\hat{p}_1)}{n_1} + \frac{\hat{p}_2(1-\hat{p}_2)}{n_2}}$$

$$upper\ bound = (\hat{p}_1 - \hat{p}_2) + z_{\alpha/2}\sqrt{\frac{\hat{p}_1(1-\hat{p}_1)}{n_1} + \frac{\hat{p}_2(1-\hat{p}_2)}{n_2}}$$

S1 Fig Standard formulae used to calculate the lower and upper bounds for the delta (difference of two proportions). Used to calculate the 95% confidence intervals for the delta change from 2018 to 2023 and from 2019 to 2023 for Ag prevalence, Mf Prevalence and the proportion of Ag-positive participants who were also Mf-positive. **Reference:** Newcombe, R.G., Interval Estimation for the Difference Between Independent Proportions: Comparison of Eleven Methods. Statistics in Medicine, 1998. 17: p. 873–890.
