# Supplementary material for: Ongoing transmission of lymphatic filariasis in Samoa 4.5 years after one round of triple-drug mass drug administration
Source: PLoS Negl Trop Dis. 2024 Jun 27;18(6):e0012236. doi: 10.1371/journal.pntd.0012236 (PMC11210818; doi:10.1371/journal.pntd.0012236)
Supplement: S1 Table — (PDF) [file pntd.0012236.s002.pdf]

Ongoing transmission of lymphatic filariasis in Samoa 4.5 years after one round of triple-drug mass drug administration

Helen J Mayfield, Benn Sartorius, Sarah Sheridan, Maddison Howlett, Beatris Mario Martin, Robert Thomsen, Rossana Tofaeono-Pifeleti, Satupaitea Viali, Patricia M Graves, Colleen L Lau

Supplementary S1 Table

S1 Table Participant numbers and demographic details for 2018, 2019 and 2023 surveys in eight sentinel primary sampling units (PSUs) in Samoa.

	2018					2019					2023				
**PSU**	**Total participants**	**Males (%)**	**Females (%)**	**Median age (min-max)**	**Houses**	**Total participants**	**Males (%)**	**Females (%)**	**Median age (min-max)**	**Houses**	**Total participants**	**Males (%)**	**Females (%)**	**Median age (min-max)**	**Houses**
**Total**	**558**	**275 (49.3%)**	**283 (50.7%)**	**24 (5-89)**	**104**	**643**	**291 (45.3%)**	**352 (54.7%)**	**20 (5-80)**	**121**	**623**	**280 (44.9%)**	**343 (55.1%)**	**22 (5-93)**	**125**
Vaivase Tai	60	35 (58.3%)	25 (41.7%)	25 (5-87)	9	58	29 (50.0%)	29 (50.0%)	24 (5-75)	15	61	30 (49.2%)	31 (50.8%)	29 (5-91)	15
Mutiatele + Saleaaumua	71	36 (50.7%)	35 (49.3%)	26 (5-70)	14	89	36 (40.4%)	53 (59.6%)	16 (5-78)	19	68	26 (38.2%)	42 (61.8%)	25 (5-77)	18
Tuanai	69	33 (47.8%)	36 (52.2%)	24 (5-79)	16	76	42 (55.3%)	34 (44.7%)	23 (5-75)	18	72	36 (50.0%)	36 (50.0%)	25 (5-93)	15
Fusi	68	31 (45.6%)	37 (54.4%)	29 (5-86)	14	69	25 (36.2%)	44 (63.8%)	21 (5-74)	12	91	46 (50.5%)	45 (49.5%)	17 (5-84)	15
Vaiusu	68	40 (58.8%)	28 (41.2%)	27 (5-75)	12	100	43 (43.0%)	57 (57.0%)	23 (5-77)	15	101	44 (43.6%)	56 (56.4%)	17 (5-78)	16
Falefa	80	39 (48.8%)	41 (51.2%)	21 5-89)	12	74	27 (36.5%)	47 (63.5%)	18 (5-77)	12	58	23 (39.7%)	35 (60.3%)	20 (5-65)	15
Faleasiu	70	28 (40.0%)	42 (60.0%)	18 (5-72)	15	102	52 (51.0%)	50 (49.0%)	16 (5-79)	15	72	29 (40.3%)	43 (59.7%)	25 (5-87)	16
Lauli'i	72	33 (45.8%)	39 (54.2%)	23 (5-81)	12	75	37 (49.3%)	38 (50.7%)	25 (5-80)	15	100	46 (46.0%)	54 (54.0%)	26 (5-73)	15
