# Supplementary material for: Ongoing transmission of lymphatic filariasis in Samoa 4.5 years after one round of triple-drug mass drug administration
Source: PLoS Negl Trop Dis. 2024 Jun 27;18(6):e0012236. doi: 10.1371/journal.pntd.0012236 (PMC11210818; doi:10.1371/journal.pntd.0012236)
Supplement: S3 Table — (PDF) [file pntd.0012236.s004.pdf]

Ongoing transmission of lymphatic filariasis in Samoa 4.5 years after one round of triple-drug mass drug administration

Helen J Mayfield, Benn Sartorius, Sarah Sheridan, Maddison Howlett, Beatris Mario Martin, Robert Thomsen, Rossana Tofaeono-Pifeleti, Satupaitea Viali, Patricia M Graves, Colleen L Lau

Supplementary S3 Table

S3 Table Microfilaria (Mf) prevalence for lymphatic filariasis in the eight sentinel primary sampling units (PSUs) in Samoa within 1.5 to 3.5 months (2018), six to eight months (2019) and 4.5 years (2023) after 1 round of triple-drug MDA. Error bars show 95% confidence intervals (CI).

	2018 (95% CI)	2019 (95% CI)	2023 (95% CI)	Change 2018 – 2023 (95% CI)	Change 2019 – 2023 (95% CI)
**Sentinel 8 PSUs**	**1.7 (0.7-3.4)**	**3.1 (1.3-5.9)**	**5.1 (1.3-12.4)**	**3.4 (1.4-5.4)**	**2.0 (-0.2-4.4)**
Vaivase tai	0.0 (0.0-4.1)	0.0 (0.0-4.2)	0.0 (0.0-4.0)	0.0 (0.0-0.0)	0.0 (0.0-0.0)
Saleaaumua + Mutiatele	0.0 (0.0-3.5)	0.0 (0.0-2.8)	0.0 (0.0-3.6)	0.0 (0.0-0.0)	0.0 (0.0-0.0)
Tuanai	0.0 (0.0-3.6)	0.0 (0.0-3.2)	0.0 (0.0-3.4)	0.0 (0.0-0.0)	0.0 (0.0-0.0)
Fusi	0.0 (0.0-3.6)	0.8 (0.1-3.4)	1.5 (0.1-6.2)	1.5 (-1.0-4)	0.7 (-2.6-4.0)
Vaiusu	4.1 (1.1-10)	2.2 (0.4-6.6)	1.3 (0-5.6)	-2.8 (-8.0-2.4)	-0.9 (-4.5-2.7)
Falefa	1.3 (0-5.6)	0.0 (0.0-3.3)	3.7 (0.8-10.2)	2.4 (-3.1-7.9)	3.7 (-1.2-8.6)
Faleasiu	1.4 (0.1-5.9)	4.4 (1.3-10.2)	11.9 (5.7-20.8)	10.5 (2.5-18.5)	7.5 (-0.1-16.0)
Lauli'i	2.7 (0.4-8.2)	7.0 (2.8-13.8)	5.5 (2.1-11.2)	2.8 (-3.0-8.6)	-1.5 (-8.8-5.8)
